# Malocclusion: Prevalence and Determinants among Adolescents of Karachi, Pakistan

**DOI:** 10.1055/s-0043-1761461

**Published:** 2023-04-14

**Authors:** Rabia Tariq, Muhammad Tahir Khan, Ashar Afaq, Sobia Tariq, Yasir Tariq, Sana Shakil Khan

**Affiliations:** 1 Department of Research, School of Public Health, Dow University of Health Sciences (DUHS), Karachi, Pakistan; 2 School of Public Health, Dow University of Health Sciences (DUHS), Karachi, Pakistan; 3 Dow International Dental College, Dow University of Health Sciences (DUHS), Karachi, Pakistan; 4 Department of Oral and Maxillofacial surgery, Liaquat College of Medicine and Dentistry, Karachi, Pakistan; 5 Center of Advanced Consultants in Healthcare Education and Training, Riyadh, Kingdom of Saudi Arabia; 6 Department of Oral and Maxillofacial surgery, Abbasi Shaheed Hospital, Karachi, Pakistan

**Keywords:** malocclusion, DMFT index, CPITN index, young adolescents, prevalence

## Abstract

**Objectives** The aim of this study was to determine the prevalence of malocclusion and its associated demographic and clinical factors in young adolescents (13–15 years) of Karachi, Pakistan.

**Materials and Methods** An epidemiological survey included 500 young adolescents of registered schools, madrassas (Islamic education system), and shop workers of Gulshan-e-Iqbal Town. It was a cross-sectional analytical study design. Multistage random sampling technique was used to enroll participants. The pattern of occlusion was recorded with other related features using Angle's classification. Health status was recorded through World Health Organization-guided indices (decayed, missing, and filled permanent teeth [DMFT], community periodontal index of treatment needs [CPITN], and body mass index [BMI]). The information, thus, obtained was analyzed through SPSS using the chi-squared test and regression models.

**Results** Forty four percent of the participants were female, while overall estimated prevalence of malocclusion in young adolescents of Karachi was 57.4%. After adjustments, participants going to any kind of education system had less malocclusion in comparison to those who were not going to any education system (adjusted odds ratio [aOR] = 0.305, 95% confidence interval [CI] = 0.12–0.73); mother's education especially higher level (aOR = 2.02, 95% CI = 1.08–3.75) and presence of periodontal disease (aOR = 1.57, 95% CI = 1.06–2.33) were significantly associated with malocclusion.

**Conclusion** This study showed that the class I malocclusion is prevalent in the local community. Demographic factors like gender, age, self-reported ethnicity, and BMI did not show any significant role. Education or knowledge of parents and young adolescents does play an influential role in decreasing malocclusion. Young adolescents, who are more prone to oral health problems at an early age, would have more chances to develop occlusal discrepancies.

## Introduction

Literature depicts that “Malocclusion is the misalignment of teeth in the upper and lower jaw due to loss of equilibrium in the relation between the hard and soft tissue of the oral cavity”. 1 According to World Health Organization (WHO) malocclusion is the third most common problem, backing dental caries and periodontal diseases among dental problems. Interestingly, this full-blown disease status of this condition has its roots in young adolescents (at the time of permanent dentition; aged 13–15 years). 2 3

Among different forms of occlusion, Angle's class I without any discrepancy in the arrangement of teeth is considered to be a normal occlusion. However, Angle's class I with the discrepancy is considered normal occlusion, while class II and class III are considered malocclusion. 4 In permanent dentition, malocclusion was globally distributed as 74.7% Angle's class I, 19.56% class II, and 5.93% class III. 5 6 7 8 In regional distribution, the prevalence of malocclusion in China and India is 45.5 and 83.3%, respectively. 9 10 The prevalence of malocclusion in Pakistan is 75.6%, which denotes a common burden on the general population. 11

Over the last decade, demographic, social, and environmental factors have also shown great influence on normal occlusion such as age, gender, low socioeconomic status, ethnicity, education, and knowledge. Although outcomes related to these factors differ globally. 12 Also, the educational status of parents and ignorance of their offspring's oral hygiene had shown a relationship with the occurrence of malocclusion traits. 13 In Italy, children and adolescents with a greater socioeconomic status had significantly lesser orthodontic treatment needs. While in the United Kingdom malocclusion was more common in deprived young adolescents. 14 A study that took place in Turkey found an insignificant association between malocclusion and demographic factors. However, related to individual clinical factors, CPITN scores had shown no significant relation with malocclusion, but DMFT scores were significant. 15 In a few Brazilian studies, national data were used in past years, to find out the relationship between sociodemographic and individual clinical factors with malocclusion occurrence. Due to conflicting results in a variety of studies, the exact part played by demographic and individual determinants remains uncertain. 12 16 17

To the best of our knowledge, in Pakistan, few studies were conducted in favor of malocclusion to assess the burden and its detriments; unfortunately, reported results are questionable when it comes to validity and generalizability. It is, therefore, imperative at assessing the burden of the condition with proper planning and implementation of survey methodology on a community level. Two hospital-based studies were conducted in 2008 and 2014 by Sakrani et al and Khan et al, respectively. They concluded that class II division-1(32%) had the highest prevalence followed by class I (29%). 18 19 Another similar study was conducted in Aga Khan Hospital Karachi, Pakistan, in 2008, by Gul-e-Erum and Fida. They concluded that class II (70.5%) and division-1 (64.7%) were the highest prevalent malocclusion among all classes. 20

Unfortunately, the lack of proper screening policies and a poorly defined general dental practice accompanied by an ill-structured optimal referral system to specialized departments and quackery (false dental practice) have led to a high burden of malocclusion in the adult population. 21 Additionally, the presence of either one or more of these causal factors can adversely affect both the physical and psychological health of an individual. Timely identification of these oral health conditions can significantly improve timely interventions, thus, reducing the overall quality of life and physical health as well as the unnecessary burden on the healthcare system. 22 23 This study aims at assessing the young adolescents (13–15 years old) of Karachi through multilevel sampling from all (registered schools, madrassas, and working young adolescence) for determining the prevalence of malocclusion based on Angle's classification and to assess the involvement of associated determinants.

## Materials and Methods

### Design and Setting

A cross-sectional study was conducted among 13 to 15 years old young adolescents of school, madrasas, and shop workers, after written approval from the Institutional Review Board (IRB) department of Dow University of Health Sciences, Karachi, Pakistan (ref# IRB-1591/DUHS/Approval/2020/83 dated March 18, 2020). The data collection was completed between the periods from April 2021 to July 2021. Two weeks before data collection, permission letters were obtained from the relevant schools, madrassas (Islamic education system), shop owners, and consent from the participant's guardian. Informed assent was also obtained directly from the participants at the time of the data collection. Young adolescents aged between 13 and 15 years with permanent dentition were included in this study.

### Sampling Technique

A multistage sampling technique was done among the nominated participants. In stage I, Gulshan-e-Iqbal Town was selected among 18 towns through judgmental sampling. It is considered to be one of the largest working-class residential and commercial neighborhoods in the East district of Karachi, Pakistan. It majorly includes all types of individuals conferred to sociodemographics.

In stage II, following proportion allocation, 24 stratified random sampling was done on Microsoft Excel software 2010 25 from the registered school and madrasas list, which was obtained from the secondary education board. Automobile shop participants were particulars through a convenience-sampling technique because of the inability to find specific shop workers and age group participants through randomization. According to gender base stratification, a total of six schools combines (male/female) and noncombine (separate males and females education system) schools were randomly nominated. Likewise, a random selection of four madrassas (two males and two females) was done.

Stage III participants were randomly selected from the sample frame of designated schools and madrassas, in accordance with the calculated sample size for each stratum. The list of nominated participants was provided in a concealed envelope to the dental examiners at the time of data collection.

### Sample Size Estimation

A sum of 284 sample population sizes was calculated with the help of “open-epi.com” by using absolute precision 5, the design effect 1.0, population size 100,0000, and anticipated frequency 75.6% as reported by the reference article. 11 This study enrolled 500 young adolescents through a fixed allocation; 350 (70%) school students in which 175 were males and 175 were females; 100 (20%) madrasa students in which 50 were males and 50 were females, and 50 (10%) working young adolescents. We inflated the sample size to 500 to accommodate for any potential selection bias. After the selection of schools and madrasas through proportion allocation, the new sample size ( *n*_new_ ) for each school and madrasah was estimated using the formula [ *n*_new_ = *n*_old_ ( *W*_k_ )]. ( *n*_old_ ) the sample size was taken according to gender-based distribution such as 175 males and 175 females for schools, and 50 males and 50 females for madrassas. ( *W*_k_ ) separate weightage was calculated by using the formula ( *W*_k_ = *N*_k_ / *N*_t_ ). 24 The total duration required to complete this study was 6 to 8 months after ethical approval.

### Data Collection

Before data collection, a training session was arranged for six dentist volunteers to discuss the objective and data collection procedure. During the pilot study, the interexaminer reliability was assessed through the Kappa coefficient for malocclusion variables ranging from 0.79 to 1.00 showing excellent reproducibility. Likewise, the interexaminer Kappa values for clinical variables of participants (body mass index [BMI] scores = 0.97, DMFT scores = 0.96, CPITN scores = 0.91) indicate strong agreement. The whole data collection process was based on three steps. On every step, two dental examiners were fixed. The first spot of data collection comprised biodata records along with height and weight measurement. On the second spot, relevant medical and dental history records and extraoral examinations were attained. Lastly, intraoral examination and oral hygiene maintenance education were done on the third spot.

### Outcomes-Related Occlusion

Regarding selected survey tool face validation was performed with the relevant subject consultants. In survey tool, extraoral features related to occlusion were recorded. The facial profile (concave or convex or straight) was recorded from the lateral view of the participant's face. In a similar glimpse, the mandibular plane angle (normal, open, and close) was also recorded. Lip competency was recorded by asking the participant to sit at ease from the frontal view. In an intraoral examination, all occlusion features were recorded. Based on the Angle's classification of the malocclusion, molar and canine relationships were recorded. 26 In cases where the molar relationship was dissimilar to the canine one, only the molar class was measured. 27 Overjet and overbite were categorized into (normal [2–3 mm], increased [>3 mm], and decreased [<2mm]). 28 Moreover, malocclusion features such as cross-bite, open-bite, Diastema, crowding, and spacing were recorded in accord with bi-variant categories (present, absent). Dental caries and periodontal status were recorded according to WHO guidelines (DMFT and CPITN indices). The DMFT score of each participant was calculated by taking the total sum of decayed, missing, and filled teeth out of 28, as per criteria. 29 Disposable dental examination instruments were used (mirror, probe, and tweezer) for dental and occlusal assessment. For periodontal assessment separately packed sterilized CPITN probe was used. Scores of 0 to 4 were recorded for six indexed teeth. Score = X was recorded in the presence of missing indexed teeth. 0= healthy gingiva, 1= bleeding on probing, 2= calculus present, 3= shallow periodontal pockets of 4–5 mm, and 4= deep periodontal pockets ≥ 6mm were scored. 30 31 Periodontal pockets were not recorded in under 15 years old young adolescents. Participants' anthropometric measures were recorded to assess their BMI of participants. Categorization of BMI factors was done according to WHO criteria. 32

### Data Analysis

All statistical analyses were performed using (SPSS) Statistical Package for Social Sciences version 24, IBM Corp, United States. Descriptive analysis was carried out for the exhibition of occlusal status according to age and gender distribution. The Pearson chi-squared (χ2) test was used to assess the associations between malocclusion and causal factors. Fisher's exact test was applied where an assumption of Pearson chi-squared test did not achieve. An advanced binary logistic regression test was conducted to calculate the odds ratio (OR) and 95% confidence interval (CI) for related factors of malocclusion. The dependent variable was malocclusion and scored as no malocclusion = 0 and malocclusion = 1. The independent variables were the individual's sociodemographic and clinical variables. The variables with ( *p* < 0.20) in the univariate analysis were tested in the multivariate binary logistic regression models. 33 Cohen's Kappa statistic was used to evaluate interexaminer variability for dental caries examination. Any values less than ( *p* < 0.05) were adopted as statistically significant for all tests.

## Result

In the current study, out of 500 young adolescents, 274 were males and 226 were females. The mean age of the participants was 14.01 ± 0.85. The overall prevalence of malocclusion in young adolescents in Karachi, Pakistan was (57.4%) that is more than half of the sample population. According to Angle's classification, the highest prevalence of malocclusion among young adolescents of Karachi, Pakistan, was class I malocclusion (43.0%), followed by class II malocclusion (10.8%) and class III malocclusion (1.4%). The prevalence of normal occlusion was equal to class I malocclusion, which is 43.2%. However, gender- and age-wise distribution of prevalence of malocclusion along with other intra- and extraoral features is given in Table 1 .

**Table 1:** Age and gender base distribution of intra- and extraoral features of young adolescents

Items	Age groups			*p* -Value	Gender		*p* -Value
	13 years *n* (%)	14 years *n* (%)	15 years *n* (%)		Male *n* (%)	Female *n* (%)	
Intraoral features							
**Malocclusion type**							
Normal occlusion	77 (35.6)	63 (29.2)	76 (35.2)	0.628 a	108 (50.0)	108 (50.0)	0.017 a
Class I malocclusion	84 (37.8)	58 (26.1)	80 (36.0)		131 (59.0)	91 (41.0)	
Class II malocclusion	16 (29.1)	14 (25.5)	25 (45.5)		28 (50.9)	17 (49.1)	
Class III malocclusion	1 (14.3)	2 (28.6)	4 (57.1)		7 (100)	0 (0)	
**Overjet**							
Normal (2–3 mm)	149 (35.2)	119 (28.1)	155 (36.6)	0.945 b	234 (55.3)	189 (44.7)	0.094 b
Increased (>3 mm)	21 (37.5)	13 (23.2)	22 (39.3)		25 (44.6)	31 (55.4)	
Decreased (<2mm)	8 (38.1)	5 (28.3)	8 (38.1)		15 (71.4)	6 (28.6)	
**Overbite**							
Normal (2–3 mm)	144 (35.4)	112 (27.5)	151 (37.1)	0.894 b	223 (54.8)	184 (45.2)	0.152 b
Increased (>3 mm)	25 (37.9)	19 (28.8)	22 (33.3)		32 (48.5)	354 (51.5)	
Decreased (<2mm)	9 (33.3)	6 (22.2)	12 (44.4)		19 (70.4)	8 (29.6)	
**Posterior cross-bite**							
Yes	4 (30.8)	3 (23.1)	6 (46.2)	0.825 a	7 (53.8)	6 (46.2)	0.944 b
No	174 (35.7)	134 (27.5)	179 (36.8)		267 (54.8)	220 (45.2)	
**Anterior cross-bite**				0.871 b			
Yes	5 (33.3)	5 (33.3)	5 (33.3)		8 (53.3)	7 (46.7)	0.908 b
No	173 (35.7)	132 (27.2)	180 (37.1)		266 (54.8)	219 (45.2)	
**Posterior open bite**							
Yes	0 (0)	2 (33.3)	4 (66.7)	0.160 a	2 (33.3)	4 (66.7)	0.417 a
No	178 (36.0)	135 (27.3)	181 (36.6)		272 (55.1)	222 (44.9)	
**Anterior open bite**							
Yes	11 (50.0)	4 (18.2)	7 (31.8)	0.329 b	12 (54.5)	10 (45.5)	0.980 b
No	167 (34.9)	133 (27.8)	178 (37.2)		262 (54.8)	216 (45.2)	
**Spacing**							
Yes	39 (40.2)	27 (27.8)	31 (32.0)	0.460 b	59 (60.8)	38 (39.2)	0.184 b
No	139 (34.5)	110 (27.3)	154 (38.2)		215 (53.3)	188 (46.7)	
**Crowding**							
Yes	44 (33.6)	34 (26.0)	53 (40.5)	0.634 b	80 (61.1)	51 (38.9)	0.093 b
No	134 (36.3)	103 (27.9)	132 (35.8)		194 (52.6)	175 (47.4)	
**Diastema** (>2mm)							
Yes	1 (33.3)	0 (0)	2 (66.7)	0.781 a	3 (100)	0 (0)	0.255 a
No	177 (35.6)	137 (27.6)	183 (36.8)		271 (54.5)	226 (45.5)	
**Extraoral features**							
**Facial profile**							
Convex	104 (37.0)	78 (27.8)	99 (35.2)	0.461 a	160 (56.9)	121 (43.1)	0.053 a
Straight	74 (34.6)	57 (26.6)	83 (38.8)		109 (50.9)	105 (49.1)	
Concave	0 (0)	2 (40.0)	3 (60.0)		5 (100)	0 (0)	
**Mandibular plane angle**							
Open	7 (41.2)	3 (17.6)	7 (41.2)	0.315 a	10 (58.8)	7 (41.2)	0.367 a
Normal	171 (35.8)	132 (27.7)	174 (36.5)		259 (54.3)	218 (45.7)	
Close	0 (0)	2 (33.3)	4 (66.7)		5 (83.3)	1 (16.7)	
**Lip competency**							
Competent	160 (35.9)	128 (28.7)	158 (35.4)	0.067 b	245 (54.9)	201 (45.1)	0.864 b
Incompetent	18 (33.3)	9 (16.7)	27 (50.0)		29 (53.7)	25 (46.3)	

a Fisher's exact test.

b Pearson chi-squared test.

The comparison of sociodemographic and clinical factors of young adolescents concerning the presence of malocclusion is given in Table 2 . Significant relationship was found between malocclusion and the level of education of young adolescents. However, 78% of young adolescents were more prevalent to malocclusion who were not attending any education system in contrast to other young adolescents who were attending any education system.

**Table 2:** Demographic and clinical characteristics of young adolescents with respect to malocclusion

Items	Malocclusion (–) *n* (%)	Malocclusion (+) *n* (%)	*p* -Value
Individual sociodemographic variables			
**Gender**			
Male	108 (39.4)	166 (60.6)	0.060
Female	108 (47.8)	118 (52.2)	
**Age**			
13	77 (43.3)	101 (56.7)	0.680
14	63 (46.0)	74 (54.0)	
15	76 (41.1)	109 (58.9)	
**Ethnicity**			
Sindhi	19 (38.0)	31 (62.0)	0.286
Pathan	29 (46.0)	34 (54.0)	
Panjabi	21 (51.2)	20 (48.8)	
Balochi	8 (26.7)	22 (73.3)	
Urdu Speaking	91 (42.1)	125 (57.9)	
Others	48 (48.0)	52 (52.0)	
**Education levels of young adolescents**			
No education	11 (22.0)	39 (78.0)	<0.001 a
School education	149 (42.6)	201 (57.4)	
Islamic education	56 (56.0)	44 (44.0)	
**Mother's education**			
Illiterate	43 (43.4)	56 (56.6)	0.090
Low	81 (46.8)	92 (53.2)	
Medium	55 (47.4)	61 (53.2)	
High	37 (33.0)	75 (67.0)	
**Father's education**			
Illiterate	26 (40.0)	39 (60.0)	0.414
Low	76 (45.0)	93 (55.0)	
Medium	44 (49.4)	45 (50.6)	
High	70 (39.5)	107 (60.5)	
** Individual clinical variables**			
**BMI**			
Underweight	92 (41.8)	128 (58.2)	0.853
Normal	103 (45.0)	126 (55.0)	
Overweight	16 (43.2)	21 (56.8)	
Obese	5 (35.7)	9 (64.3)	
**Dental caries**			
Absent	123 (48.4)	131 (51.6)	0.017 a
Present	93 (37.8)	153 (62.2)	
**Early loss of permanent teeth**			
Absent	211 (44.0)	269 (56.0)	0.094
Present	5 (25.0)	15 (75.0)	
**Periodontal disease**			
Absent	131 (49.4)	134 (50.6)	0.003 a
Present	85 (36.2)	150 (63.8)	

Abbreviation: BMI, body mass index.

Pearson chi-squared test.

a *p* -Value < 0.05 is significant.

Moreover, young adolescents with malocclusion were more prone to develop caries than young adolescents without malocclusion. Results had shown a significant relationship between malocclusion with decayed teeth, while the relationship with the early loss of permanent teeth due to caries was insignificant. Young adolescents with malocclusion (63.8%) were significantly found to suffer from periodontal problems, while 50.6% of young adolescents with malocclusion had healthy gums.

Moreover, advanced univariate logistic regression was used to assess the significant factors for malocclusion status, as shown in Table 3 . The odds of malocclusion status among females were 0.71 times lesser compared to the odds of malocclusion among males (OR = 1.40, 95% CI = 0.98–2.00). Young adolescents aged 14 years were 0.89 times less likely to have malocclusion status than 13 and 15 years, but it was not significant ( *p* = 0.629, 95% CI = 0.57–1.40). The odds of malocclusion among young adolescents in school were 0.38 times lower (OR = 0.38, 95% 95% CI = 0.18–0.76) and the odds of malocclusion among young adolescents of madrassas (Islamic education system) were 0.22 times lower (OR = 0.22, 95% CI = 0.10–0.48) compared to odds of malocclusion among young adolescents of shop workers, who did not go to any type of education system. Furthermore, the factors ( *p* < 0.20), which were statistically significant, were added in a single multivariate model 33 to ascertain the effect of factors on the likelihood that young adolescents have a malocclusion. Young adolescents with periodontal diseases were 1.57 times more likely to have malocclusion as compared to young adolescents without periodontal diseases, and it was significant ( *p* = 0.022, 95% CI = 1.06–2.33). However, in univariate logistic regression analysis, the young adolescents with dental caries were 1.54 times more likely to have malocclusion than those who did not have dental caries ( *p* = 0.017, 95% CI = 1.08–2.20). Still it became insignificant with other covariant in multivariate logistic regression analysis.

**Table 3:** Univariate and multivariate logistic regression for identifying significant factors of malocclusion

Items	Univariate			Multivariate		
	*p* -Value	OR	95% CI	*p* -Value	aOR	95% CI
Individual sociodemographic variables						
**Age groups**						
13		Reference				
14	0.629	0.895	0.57–1.40			
15	0.675	1.093	0.72–1.65			
**Gender**						
Male		Reference			Reference	
Female	0.060 a	0.711	0.49–1.01	0.286	0.806	0.54–1.19
**Ethnicity**						
Sindhi		Reference				
Pathan	0.392	0.719	0.33–1.53			
Panjabi	0.208	0.584	0.25–1.34			
Balochi	0.302	1.68	0.62–4.53			
Urdu speaking	0.593	0.842	0.44–1.58			
Others	0.247	0.664	0.33–1.32			
**Education levels of young adolescents**						
No education		Reference			Reference	
School education	0.007 a	0.38	0.18–0.76	0.060	0.455	0.20–1.03
Islamic education	<0.001 a	0.22	0.10–0.48	0.008 b	0.305	0.12–0.73
**Mother's education**						
Illiterate		Reference			Reference	
Low	0.59	0.87	0.53–1.43	0.97	0.99	0.57–1.69
Medium	0.55	0.85	0.49–1.46	0.64	1.14	0.63–2.07
High	0.12 a	1.55	0.89–2.72	0.026 b	2.02	1.08–3.75
**Father's education**						
Illiterate		Reference				
Low	0.49	0.81	0.45–1.45			
Medium	0.24	0.68	0.35–1.30			
High	0.94	1.01	0.57–1.82			
** Individual clinical variables**						
**BMI**						
Underweight		Reference				
Normal	0.50	0.87	0.60–1.27			
Overweight	0.87	0.94	0.46–1.90			
Obese	0.65	1.29	0.42–3.98			
**Dental caries**						
Absent		Reference			Reference	
Present	0.017 a	1.54	1.08–2.20	0.62	1.35	0.44–4.53
**Early loss of permanent teeth**						
Absent		Reference			Reference	
Present	0.103 a	2.353	0.84–6.57	0.12	2.67	0.75–9.45
**Periodontal disease**						
Absent		Reference			Reference	
Present	0.003 a	1.72	1.20–2.47	0.022 b	1.57	1.06–2.33

Abbreviations: aOR, adjusted odds ratio; BMI, body mass index; CI, confidence interval.

a *p* -Value < 0.20 is significant for univariate logistic regression analysis.

b *p* -Value< 0.05 is significant for multivariate regression analysis.

## Discussion

This epidemiological study involved 500 (13–15 years) young adolescents of school, madrassas, and shop workers in Gulshan-e- Iqbal Town, Karachi, Pakistan, who were selected to provide the burden of malocclusion and significantly related demographic and local factors that seem to be the common oral health concern in our early population. To meet the major groups of the target population, a technical stage III sampling concept was proposed.

In the current study, the total prevalence of malocclusion in permanent dentition was 57.4%. In Africa, research on the prevalence of malocclusion in young adolescents showed a difference ranging from 63.8% in Tanzania to 95.6% in Libya, 34 35 according to National Health and Nutrition Estimates Survey III. In Asia, the prevalence was 86.7% in Iran, 36 India at 77.1%, 37 and China at 47.92%. 38 In Romania, the overall prevalence of malocclusion was 93.5% 39 that is almost similar to the United States. 40

According to our information, in Pakistan, most of the studies on the prevalence of malocclusion were hospital-based, 19 20 41 which are uncertain in terms of general population estimates. Nevertheless, few schools-based studies have reported a prevalence of malocclusion ranging from 73.2 to 75.6%. 11 42 The probable high prevalence of malocclusion might be due to different study parameters and targeting specific population groups, rather than targeting the general population by Abbas et al. 11 Similarly, the study conducted by Nazir et al 43 targeted the vulnerable population. The possibility of limited resources and lack of good oral hygiene maintenance for a longer duration at an early age could inflate the prevalence of malocclusion. 42

The discrepancies in the overall malocclusion outcome frequencies indicate that the prevalence of malocclusion varies based on gender, age, variation in ethnicity, a form of malocclusion, geographic regions, provinces, number of years, genetic predisposition, the cross-cultural difference in living standards, growth variance, development of the facial skeleton, and even diagnostic criteria. Malocclusion is widely considered a major public health concern, and its underlying causes are multifactorial such as biological, race, developmental factors, oral behaviors, and environmental factors.

In our analysis, the class I molar relationship with normal occlusion was 43.2% that was almost equivalent to class I malocclusion (43%) followed by class II malocclusion (10.8%) and class III malocclusion (1.4%), according to Angle's classification. The global distribution of class I was lowest in Europe (60.3%) and highest in Africa (83.6%), while class II distribution was highest in Europe (33.5%) and lowest in Africa (11.4%). The distribution of class III was approximately the same (6.3%) in all continents except Africa, which was (4.7%). 44 Class I malocclusion prevalence was approximately identical to that of Serbia (43.3%) 45 and Albania 40.4%. 46 Bilgic et al recorded a 34.9% prevalence in Turkey, 5 which was lower than the current study. However, a higher prevalence of class I malocclusion was reported by Gudipaneni et al registered in KSA 52.8%, 47 Silva and Kang in United States 62.9%, 40 and Singh 87.4% in India. 48 In this analysis, the distribution of class II malocclusion was 10.8%, almost equivalent to studies performed by Singh, that is, 10.1%, 48 and Hasan SM 49 , that is, 9.34%. Nevertheless, Gudipaneni et al, 47 Borzabadi-Farahani et al, 6 and Laganà et al 46 registered a prevalence of 27.5% to 31.8%, which was high in this sample. In comparison to other studies, a lower incidence of class III malocclusion (1.4%) was found in the current report, varying from 2 to 17.9%. 34 40 50 51 Aikins and Onyeaso, however, reported an approximately similar prevalence of (1.6%) class III malocclusion. 52

From Pakistan, Nazir et al registered a 48.6% class I prevalence, which was marginally higher than the current investigation. The comparison of the findings shows that the current study was performed in populations with different sample groups (adolescents of schools, madrassa, and shop workers), ethnicity, culture, and region. In addition, Khan et al 19 and Sakrani et al 18 reported the higher prevalence of class II (37.2%, 56%) and class III (12.5%, 4%) respectively. The disparity in outcome possibly reflects the difference between the hospital-based study design and the sample population.

The additional occlusal discrepancies like hypodontia, impacted canines, malformed laterals, transposition, and supernumerary teeth were often reported with class II and class III malocclusion. The association between craniofacial discrepancies and congenital anomalies may generate complicated therapeutic problems. However, factors like congenital anomalies, which are leading to craniofacial discrepancies, are still under debate. 53 54 Moreover, as an etiology, malocclusion correlates with multiple variables. Local factors and sociodemographic variables were significant highlights of the current research. Sociodemographic factors are of great concern at an early stage of life, particularly in developing countries, where the illiteracy rate is more than half, as in Pakistan. 55 As a consequence, the effect of rising economies and enlarged expenditures has been attributed to lifestyle changes. Finally, each family member, including children and young adolescents, will bear the influence of all expenditure loads. At the same time, exposure to multiple factors possibly predisposes the malocclusion at an early age (deciduous dentition) that eventually impacted occlusion and overall oral health far ahead (permanent dentition). 56 57 Proportionate groups were taken to include general young adolescents from the Pakistani community (school-going young adolescents, religious education (madrassas)-going young adolescents, and young adolescents working in shops. To the best of our knowledge, no comparative finding was reported based on the education levels of young adolescents other than in private and public schools that malocclusion was more prevalent in young adolescents in public schools. 58 In the current study, young adolescents of school and madrassas were more likely to have malocclusion status in contrast to illiterate young adolescents as shop workers with significant logistic regression analysis (adjusted OR [aOR] = 0.455, 95% CI = 0.20–1.03) (aOR = 0.305, 95% CI = 0.12–0.73), respectively.

In developing countries, maternal education is of paramount importance for a nation's development, together with individuals themselves, separately within the group. This study had shown a higher prevalence rate of malocclusion in young adolescents of poorly educated mothers and highly educated mothers, as compared to moderately educated mothers that were even higher in frequency. The possible interpretation of two extreme-level outcomes was a lack of knowledge and a lack of attention to oral health at a young age. Similarly, Tsasan Tumurkhuu et al reported the independent relation of malocclusion with maternal education, that is, the prevalence of malocclusion was higher in young adolescents of higher-educated mothers. 59

A strong point of this study was the use of the investigative approaches suggested by the WHO (CPITN and DMFT). 31 60 Both indices are considered valid and reliable, therefore leading to a low level of measurement bias. The results had shown a significant relation between CPITN scores with malocclusion in the current study. Dental caries was associated with malocclusion in the chi-squared test. However, in binary logistic regression analysis, caries and early loss of permanent teeth due to caries or extraction due to caries had shown a strong association with malocclusion, but it was insignificant with other variants. Consequently, missing permanent teeth may lead to disruption of the normal alignment of teeth. Moreover, the likelihood of dental caries, along with a lack of attention to the need, suggests that the condition worsens and eventually contributes to the need for more complex treatments. This requirement is not limited to the people we studied only but also affects the general population of other regions, as reflected in the data of other studies. 61 62 A similar association had stated in another study by Abbas et al from Pakistan. 11 In addition to dental caries, periodontal health is also interconnected with other oral health problems including malocclusion reported in other studies. 63 64 This dependency on periodontal health would be grounded on the foundation of the type and severity of the malocclusion. Moreover, systemic illness or neurodevelopmental disorders may compromise oral health status at an early age due to negligence. This may aggravate the vicious circle of common oral health problems. 65

Our study has some strengths and limitations; this is the only study employing a multistage sampling technique to target the general population for the assessment of malocclusion and its associated factors in Pakistan. A predesigned survey tool considering WHO-guided indices and effective methodology with low technological and technical support requirements allowed us to obtain relevant data from the target population with a low risk of bias. The demographic factors were self-reported by the participants and due to the same reason a socioeconomic and family history-related factors of the target population were not measured to avoid bias.

## Conclusion

Malocclusion is a widespread phenomenon and class I malocclusion is the most prevalent among 13 to 15 years old young adolescents. Inequalities in its distribution are determined by the types of education systems of young adolescents and the mother's level of education. Malocclusion has a strong association with local environmental factors like the periodontal health of young adolescents. It can be controlled by detecting the problems early and treating them one by one.
